# Association between human papillomavirus particle production and the severity of recurrent respiratory papillomatosis

**DOI:** 10.1038/s41598-023-32486-8

**Published:** 2023-04-06

**Authors:** Satoshi Yamada, Toshiya Itoh, Taro Ikegami, Atsushi Imai, Daiki Mochizuki, Hiroshi Nakanishi, Ryuji Ishikawa, Junya Kita, Yuki Nakamura, Yoshinori Takizawa, Jun Okamura, Yoshihiro Noda, Toshihide Iwashita, Takahiko Hariyama, Mikio Suzuki, Kiyoshi Misawa, Hideya Kawasaki

**Affiliations:** 1 Department of Otolaryngology/Head and Neck Surgery, Hamamatsu University School of Medicine, 1-20-1 Handayama Higashi-ku, Hamamatsu, Shizuoka 431-3192 Japan; 2 Preeminent Medical Photonics Education and Research Center Institute for NanoSuit Research, Hamamatsu University School of Medicine, 1-20-1 Handayama Higashi-ku, Hamamatsu, Shizuoka 431-3192 Japan; 3 Department of Obstetrics and Gynecology, Hamamatsu University School of Medicine, Hamamatsu, Japan; 4 Department of Otorhinolaryngology, Head and Neck Surgery, Graduate School of Medicine, University of the Ryukyus, Okinawa, Japan; 5 Department of Otorhinolaryngology, Seirei Hamamatsu General Hospital, Hamamatsu, Japan; 6 Department of Otorhinolaryngology, Seirei Mikatahara General Hospital, Hamamatsu, Japan; 7 Department of Regenerative and Infectious Pathology, Hamamatsu University School of Medicine, Hamamatsu, Japan

**Keywords:** Human papilloma virus, Viral infection, Predictive markers

## Abstract

Recurrent respiratory papillomatosis (RRP) has a wide range of severity. We investigate the relationship between human papillomavirus (HPV) particle production and severity of RRP. From September 2005 to June 2021, 68 RRP samples (from 29 patients) were included. HPV type was determined. HPV viral load, physical status, and demographic and clinical characteristics were assessed. Immunohistochemistry (IHC) was performed for p16, Ki-67, L1, and E4. We used NanoSuit-CLEM (correlative light and electron microscopy) and transmission electron microscopy (TEM) to examine the samples. The total number of surgeries in HPV-positive and HPV-negative cases were 3.78 (n = 55/68, range: 1–16) and 1.30 (n = 13/68, range: 1–3), respectively (*p* = 0.02). IHC showed that L1 and E4 were correlated and expressed on the tumour surface. NanoSuit-CLEM and TEM revealed HPV particles in L1-positive nuclei. L1 IHC-positive cases had a shorter surgical interval (*p* < 0.01) and more frequent surgeries (*p* = 0.04). P16 IHC, viral load, and physical status were not associated with disease severity. This study visualised HPV particle production in RRP for the first time. Persistent HPV particle infection was associated with severity. We suggest L1 IHC for evaluating RRP severity in addition to the Derkay score.

## Introduction

Recurrent respiratory papillomatosis (RRP) is a benign tumour that is often caused by human papillomavirus (HPV) type 6 or 11^1,2^. RRP is also known as laryngeal papillomatosis (LP)^3^. In general, HPV involvement is reported in 80–96.9% of patients^4–6^; however, a few studies reported lower rate of HPV detection approximately 60%^1,2^. Moreover, some reports have not examined HPV involvement^7–9^. Some patients remain in remission after a single surgery, while others develop malignant transformation after multiple surgeries and have unfavourable outcomes^10,11^. The Derkay score, which evaluates disease severity based on clinical findings, is often used to determine the severity of RRP^12^. However, few studies have been conducted to investigate the clinical severity of the disease based on its pathogenesis, such as the HPV lifecycle. Since HPV DNA is detected in the laryngeal mucosa of patients in remission, it is believed that persistent HPV infection is involved in the pathogenesis of RRP^13^. However, no reports have examined the association between HPV involvement (or the state of HPV particles produced by tumours) and disease severity.

The NanoSuit method is a biomimetic technique inspired by the ability to observe live *Drosophila* larvae using a field emission scanning electron microscope (FE-SEM), which requires high vacuum conditions^14,15^. The NanoSuit-CLEM (correlative light and electron microscopy) method, which applies NanoSuit technology, is a technique that forms a nano membrane on pathological tissues, thereby preserving the water-content of the tissue and maintaining its three-dimensional structure, while the area under optical microscopic observation is observed with a SEM^16^. The process can be completed in a few minutes, and FE-SEM observation can be easily performed. We previously examined various pathological tissues such as the gastrointestinal tract and salivary gland using the NanoSuit-CLEM method^17–19^. Furthermore, we have previously reported standard methods of observation for various viruses, such as cytomegalovirus, varicella zoster virus, and HPV, using the NanoSuit-CLEM method^20^.

It has been reported that HPV E4 and E5 mRNA expressions are high in RRP and may be involved in the pathogenesis of the disease^21,22^. Thus, we hypothesised that active HPV particle production would be associated with the severity of RRP because E4 is involved in the formation and release of HPV particles.

In this study, we attempted to elucidate the HPV lifecycle in RRP by observing HPV particles using the NanoSuit-CLEM method. Furthermore, we aimed to show that active HPV particle production is associated with disease severity.

## Results

### Patient characteristics

The patient characteristics are summarised in Table 1 and detailed data are available in Supplementary Table S1. We performed direct sequencing analysis to determine the HPV type (Supplementary Fig. S1). The mean age was 44.83 (range: 30–69, SD: 8.67), 57.00 years (range: 45–69, SD: 12.53), and 60.10 (range: 37–86, SD: 16.04) years in patients with HPV type 6, 11, and negative, respectively. All the cases were adult onset RRP, and treatment was performed in the operating room under general anaesthesia. None of the patients received HPV vaccination. Patients with RRR were mostly male (n = 25/29, 86.2%), had a history of smoking (n = 23/29, 79.3%), and had a history of alcohol consumption (n = 24/29, 82.8%). The number of total surgeries was 3.78 (n = 19/29, range: 1–16) in HPV-positive cases, and 1.30 (n = 10/29, range: 1–3) in HPV-negative cases (*p* = 0.02). Furthermore, the Derkay score was 6.27 (n = 55/68, range: 3–14) in HPV-positive cases, and 4.84 (n = 13/68, range: 3–8) in HPV-negative cases (*p* = 0.02). However, no statistically significant differences in the total number of surgeries and Derkay scores were identified for HPV types 6 and 11 (*p* = 0.10). No cases of disseminated lesions in the lower respiratory tract were observed. Malignant transformation occurred in three cases, all of which were HPV-negative.

**Table 1:** Patient characteristics.

	HPV (+)	HPV (−)	*p*	HPV 6	HPV 11	*p*
Sex						
M	16	9		10	6	
F	3	1	0.62^a^	2	1	0.77^a^
Age mean (range, SD)	49.31 (30–69, 11.60)	60.10 (37–86, 16.04)	0.08^b^	44.83 (30–58, 8.67)	57.00 (45–69, 12.53)	**0.04**^b^*
Smoking						
+	15	8		9	6	
−	4	2	0.68^a^	3	1	0.55^a^
Alcohol						
+	16	8		10	6	
−	3	2	0.81^a^	2	1	0.77^a^
HPV vaccine	0	0	−	0	0	−
No. of total surgeries	3.78 (1–16)	1.30 (1–3)	**0.02**^b^*	3.36 (1–8)	4.43 (1–16)	0.67^b^
Derkay score	6.27 (3–14)	4.84 (3–8)	**0.02**^b^*	5.68 (3–10)	6.92 (3–14)	0.10^b^
Malignant transformation						
+	0	3			0	
−	19	7	**0.03**^a^*	12	7	−

Significant values are in [bold].

*HPV* Human papillomavirus, *M* Male, *F* Female, *SD* Standard deviation.

*Indicates *p* < 0.05. Data for the two populations in the two categories are subjected to a Fisher’s exact test. A Welch’s t-test is applied to compare two sets of data.

^a^Indicates Fisher’s exact test.

^b^Indicates Welch’s t-test.

### Pathological findings

**Figure 1:**
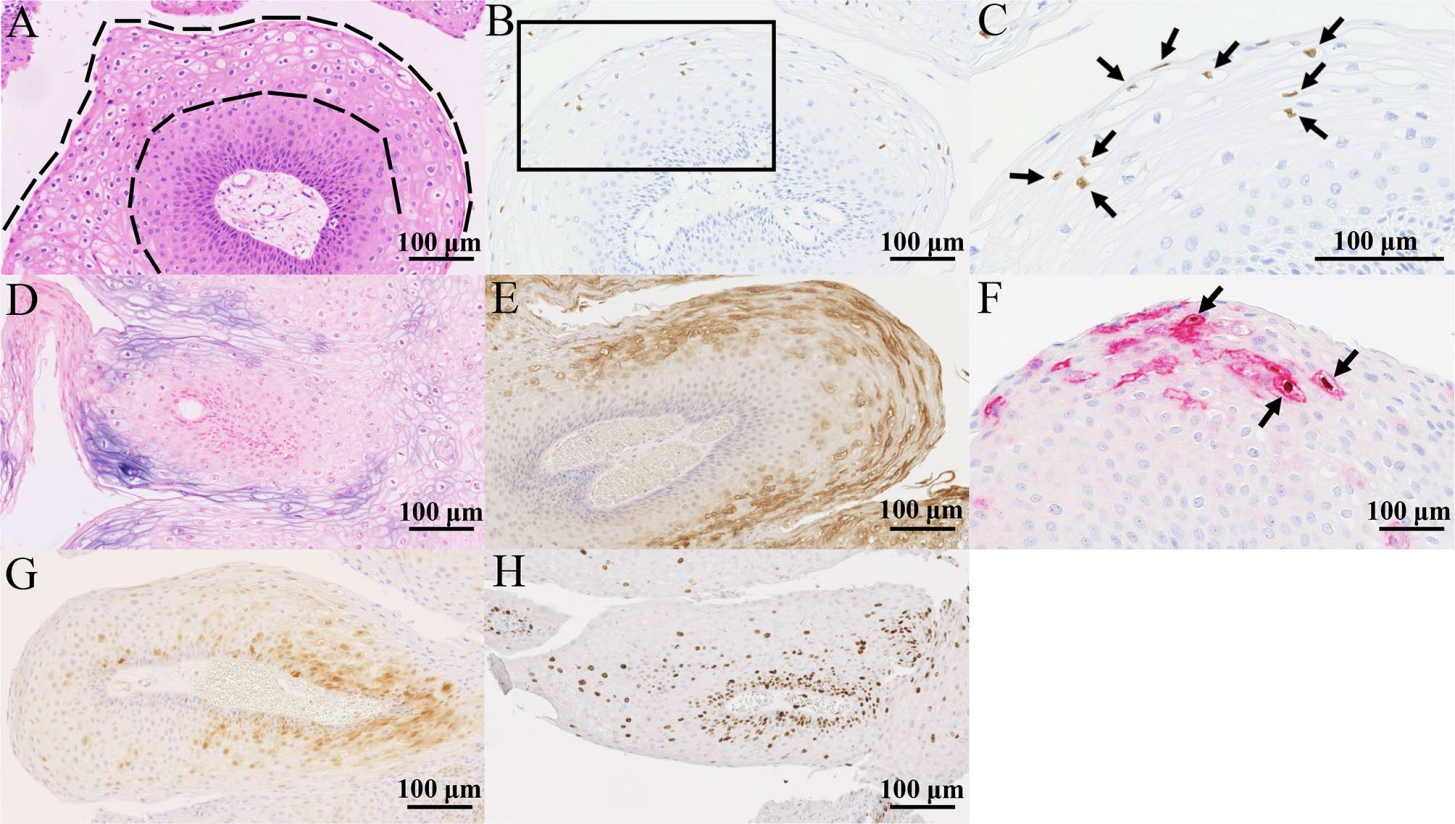
Pathological findings. (**A**) A koilocytotic lesion with nuclear atypia and perinuclear vacuoles is observed on the tumour surface (area between the dotted lines). (**B**,**C)** [(**C**) is an enlargement of the square in (**B**)]: HPV L1 IHC showing positive cells in tumour surface koilocytotic lesions (arrow). (**D**) RNA-in situ hybridization of HPV E4 expressed cell membrane in tumour surface koilocytotic lesions. (**E**) HPV E4 IHC and RNA-in situ hybridization are positive for HPV E4 for the same lesion. (**F**) HPV L1 and HPV E4 are expressed in a similar distribution on the tumour surface, with some sites of both being positive in the same cell (arrow). Brown indicates HPV L1 and red indicates HPV E4. (**G**) P16 IHC shows weak dispersion on staining. (**H**) Ki-67 IHC staining is mainly observed in the basal layer, with the percentage of positive cells decreasing towards the tumour surface. Only (**C**) is observed at a 400 × field of view, while the others are observed at a 200 × field of view. Bars indicate 100 μm. *HPV* Human papillomavirus, *IHC* Immunohistochemistry.

HPV-positive RRP cases often showed koilocytosis on the tumour surface (area between the dotted lines) (Fig. 1A). On the surface of the tumour, mainly in koilocytotic lesions, the nucleus was positive for L1 immunohistochemistry (IHC), which is the capsid protein of HPV, and the cell membrane was positive for E4 mRNA and protein, which are involved in HPV particle formation and release (Fig. 1B–E). Both L1 and E4 are expressed in a similar distribution on the tumour surface, with some sites of both being positive in the same cell (arrow) (Fig. 1F). P16 IHC showed weak dispersion on staining (Fig. 1G). Ki-67 staining was mainly observed in the basal layer, with the percentage of positive cells decreasing toward the tumour surface (Fig. 1H).

### Histological markers and HPV involvement

**Figure 2:**
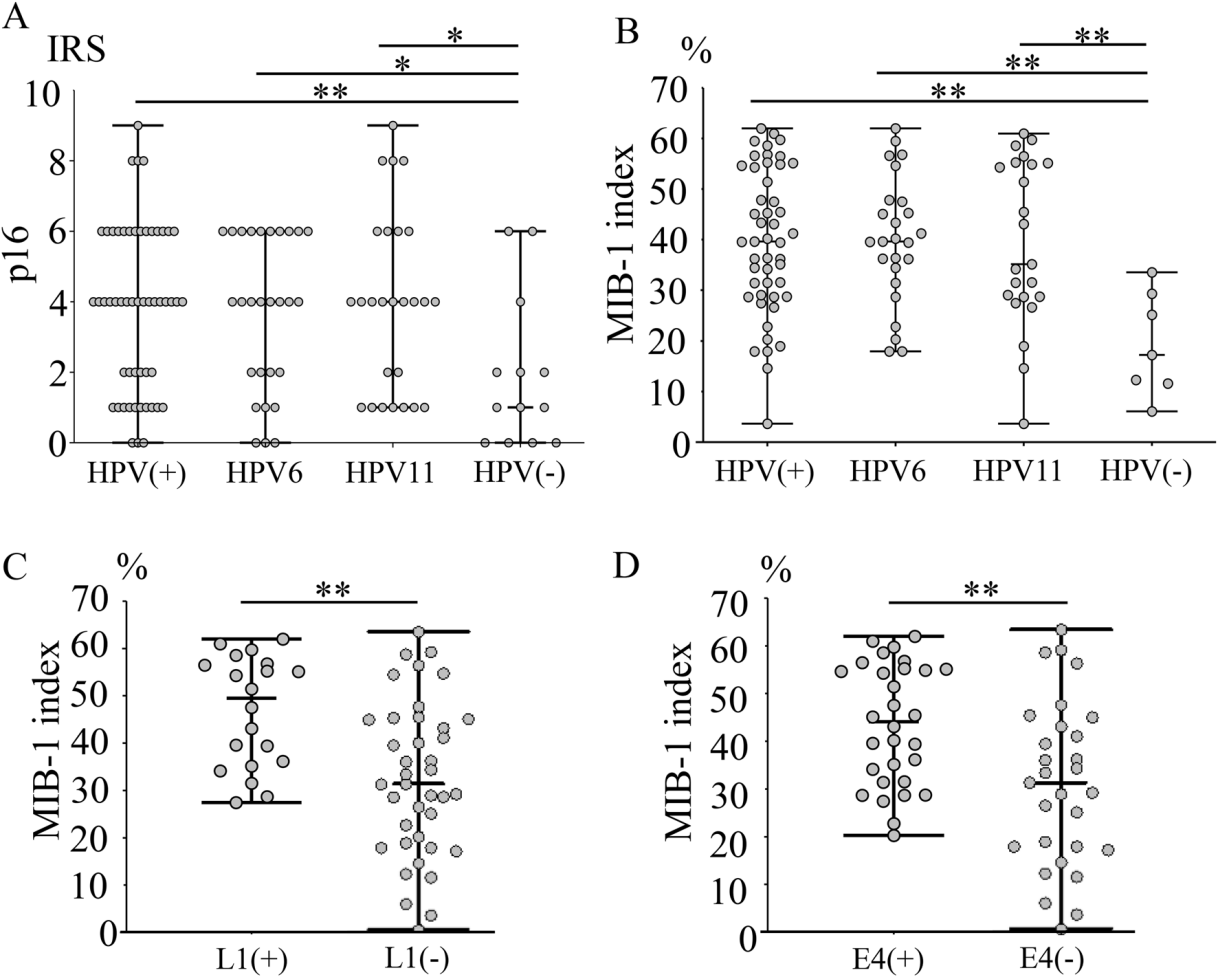
Quantitative evaluation of pathological findings. (**A**) P16 staining is statistically significant in HPV-positive cases, HPV type 6-positive cases, and HPV type 11-positive cases compared to negative cases (*p* < 0.01, *p* = 0.02, and *p* = 0.02, respectively). (**B**) The MIB-1 index is statistically significant in HPV-positive cases, HPV type 6-positive cases, and HPV type 11-positive cases compared to negative cases (all *p* < 0.01). (**C**) MIB-1 index is significantly higher in HPV L1-positive cases than in negative cases (*p* < 0.01). (**D**) MIB-1 index is statistically significant in HPV E4-positive cases than in negative cases (*p* < 0.01). * indicates *p* < 0.05 and ** indicates *p* < 0.01. *HPV* Human papillomavirus.

We used immunoreactive score (IRS) to evaluate p16 IHC staining (Supplementary Fig. S2). P16 IRS was statistically significant in the HPV-positive cases compared to those in the negative cases (*p* < 0.01). However, there was no statistically significant difference between HPV types 6 and 11 (*p* = 0.62). (Fig. 2A). MIB-1 index was significantly higher in HPV-positive cases than in HPV-negative cases (*p* < 0.01). However, there was no statistically significant difference between HPV types 6 and 11 (*p* = 0.87) (Fig. 2B). Comparing L1 IHC-positive and -negative cases, the MIB-1 index was statistically higher in the positive cases (*p* < 0.01) (Fig. 2C). Similarly, E4 IHC was statistically higher in the HPV-positive cases (*p* < 0.01) (Fig. 2D).

### HPV particle detection

**Figure 3:**
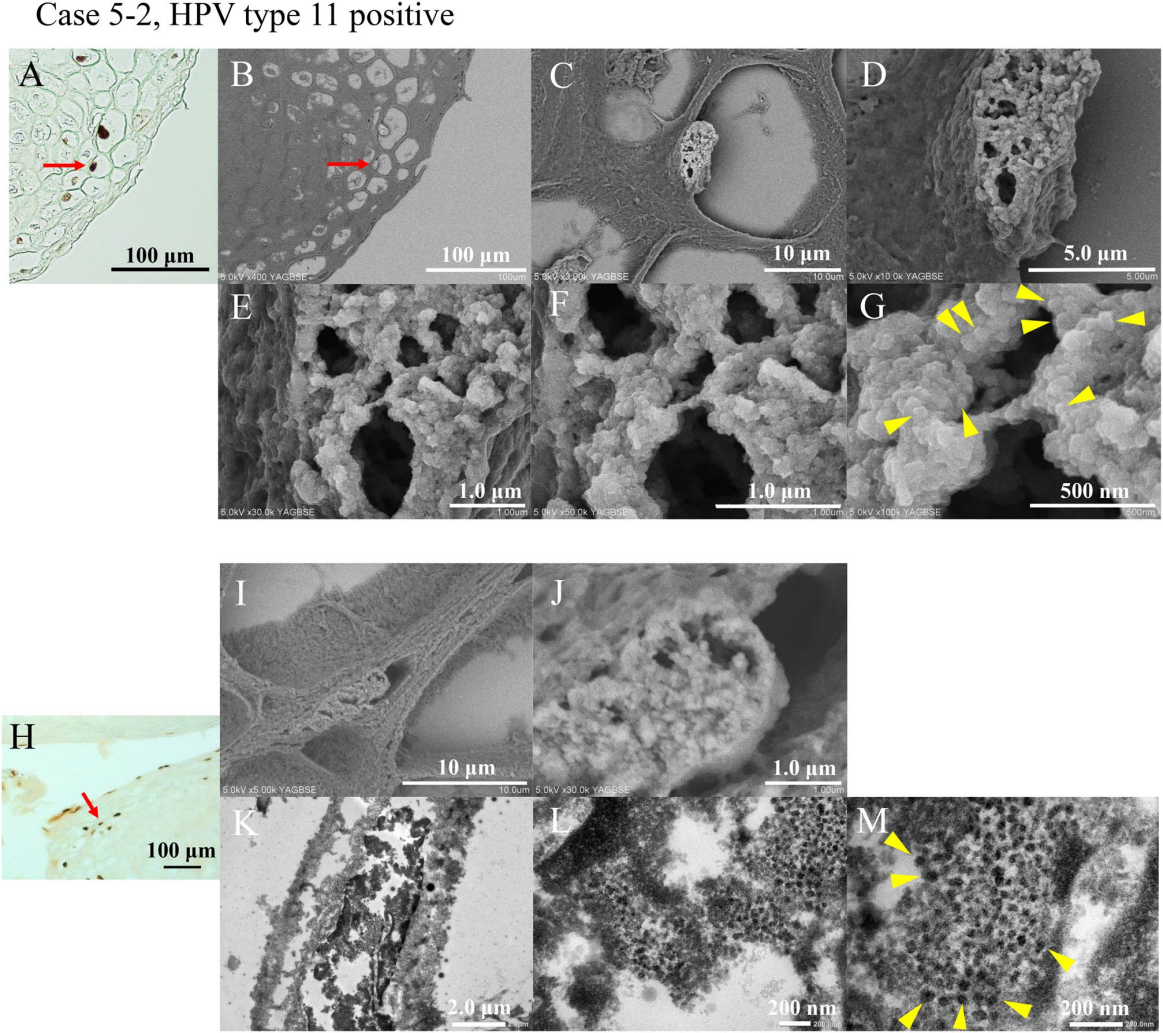
HPV particle detection in HPV L1-positive cells. (**A**) HPV L1 IHC is performed, and the arrow indicates the observation object. Cell nuclei are indicated by arrows. A 400 × field of view is observed. Bar indicates 100 μm. (**B**–**G**) HPV L1 IHC-positive cell nuclei are observed using the NanoSuit-CLEM method. The nuclei are filled with the microparticles. Typical particles of approximately 50–60 nm, the same size as the HPV particles, are indicated by arrowheads (**G**). The field of view of the images are 500 × (**B**), 3000 × (**C**), 10,000 × (**D**), 30,000 × (**E**), 50,000 × (**F**), and 100,000 × (**G**). The bars indicate 100 μm (**B**), 10 μm (**C**), 5.0 μm (**D**), 1.0 μm (**E**), 1.0 μm (**F**), and 500 nm (**G**). (**H**) HPV L1 IHC is performed. The arrow indicates the observation object. The field of view is 200 × . Bars indicate 100 μm. (**I**, **J**) HPV L1 IHC-positive cell nuclei are observed using the NanoSuit-CLEM method. The nuclei are filled with the microparticles. The fields of view are 5000 × (**I**) and 30,000 × (**J**). Bars indicate 10 μm (**I**) and 1.0 μm (**J**). (**K**–**M**) The areas are observed by the NanoSuit-CLEM method using TEM. Microparticles of approximately 50–60 nm in size are observed in the nucleus and determined to be HPV particles. Representative HPV particles are indicated with arrowheads (**M**). Magnification is 5000 × (**K**), 30,000 × (**L**), and 50,000 × (**M**). The bars indicate 2.0 μm (**K**), 200 nm (**L**), and 200 nm (**M**). *CLEM* Correlative light and electron microscopy, *HPV* Human papillomavirus, *IHC* Immunohistochemistry.

We attempted to identify HPV particles in formalin-fixed paraffin-embedded (FFPE) sections. We selected Case 5–2, who was HPV type 11-positive and well expressed L1 IHC. First, we performed L1 IHC and photographed the positive sites (Fig. 3A). Subsequently, 1% osmium incubation and osmium vapor deposition were performed. The arrow section of the L1 IHC-positive nucleus was identified using FE-SEM (Fig. 3B). L1-positive cells were enhanced by the reaction of osmium with 3–3′-diaminobenzidine (DAB). Observation of the nucleus at increasing magnification revealed the presence of numerous microparticles (Fig. 3C–G). Typical particles of approximately 50–60 nm, the same size as the HPV particles, are indicated by arrowheads (Fig. 3G).

Next, we attempted TEM observations of the microparticles in the nucleus using FE-SEM. IHC for L1 was performed (Fig. 3H), NanoSuit solution II was applied, and the sample examined using FE-SEM. The L1-positive cell nuclei were filled with microparticles (Fig. 3I,J). Next, the observed tissue on the glass slides was embedded in epoxy resin, and ultrathin specimen sections were prepared and observed by TEM. The same nuclei observed by FE-SEM were also observed using TEM. Numerous microparticles of approximately 50–60 nm were found in the nuclei of L1-positive cells (Fig. 3K–M). Thus, we concluded that the particles in the L1-positive nuclei were HPV particles.

### Visualisation of the HPV particle formation process

**Figure 4:**
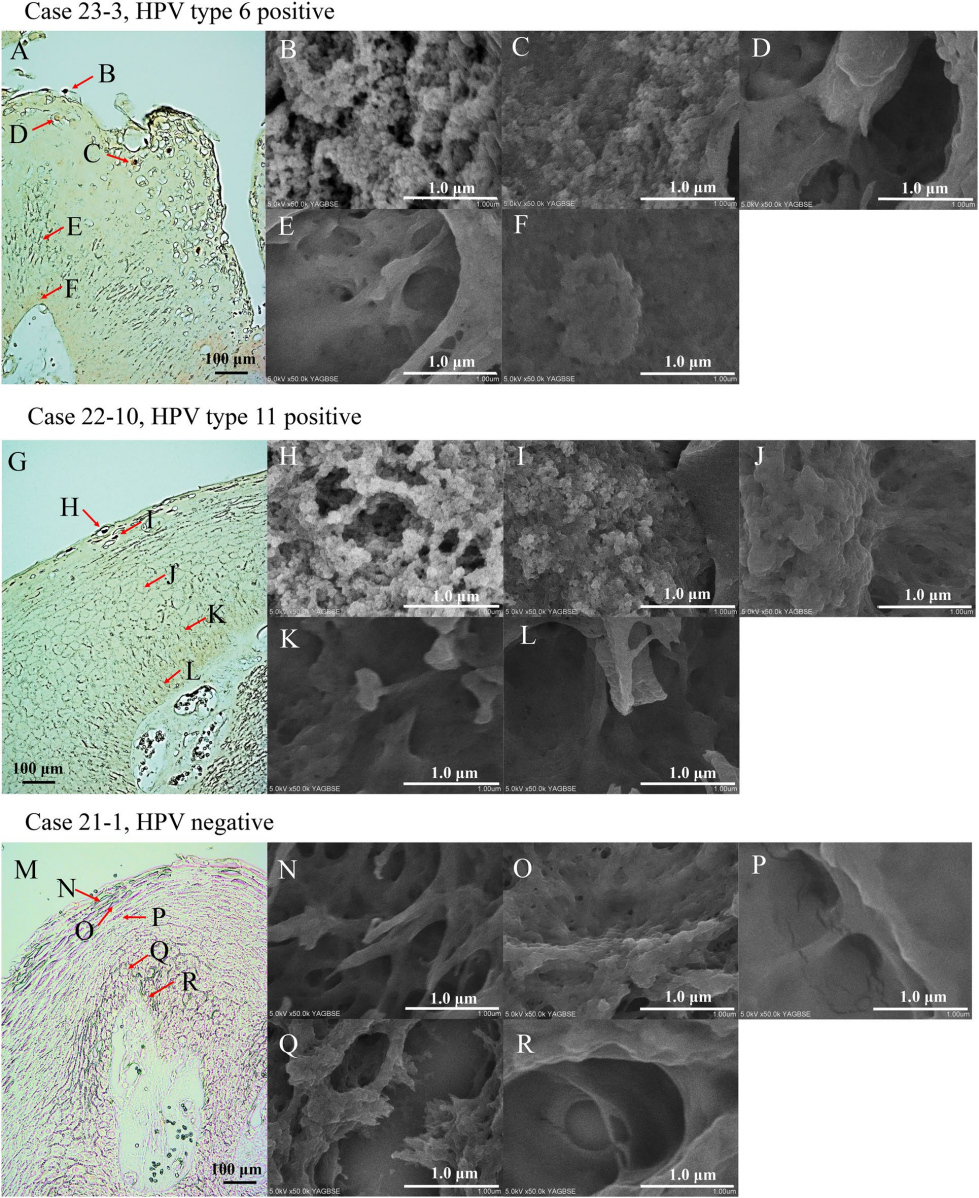
HPV particle formation process. (**A**–**F**) The process of particle formation in the HPV type 6 case. HPV L1 IHC is performed, and the arrows indicate the observation object. A 200 × field of view is observed. The bar indicates 100 μm (**A**). In areas where HPV L1 IHC is strongly stained (**B**), numerous HPV particles are observed, whereas in areas where HPV L1 IHC is weakly stained (**C**), the number of HPV particles is reduced, and some particles appear to be immature. Areas where HPV L1 IHC-negative lesions show no HPV particles (**D**–**F**). At 50,000 × field of view, the white bar indicates 1.0 μm (**B**–**F**). (**G**–**L**) The process of particle formation in HPV type 11 cases. HPV L1 IHC is performed, and the arrows indicate the observed object. A 200 × field of view is observed. The black bar indicates 100 μm (**G**). In areas where HPV L1 IHC is strongly stained (**H**), numerous HPV particles are observed, whereas in areas where L1 is weakly stained (**I**), the number of HPV particles is reduced, and some particles appear to be immature. Areas where HPV L1 IHC-negative lesions show no HPV particles (J–L). At 50,000 × field of view, the white bar indicates 1.0 μm (**H**–**L**). (**M**–**R**) HPV-negative case. HPV L1 IHC is performed, and the arrows indicate the observed object. A 200 × field of view is observed. The black bar indicates 100 μm (**M**). No HPV particles are observed (N–R). At 50,000 × field of view, the white bar indicates 1.0 μm (**N**–**R**). *HPV* Human papillomavirus, *IHC* Immunohistochemistry.

In addition, we attempted to determine whether HPV particles appear differently in each HPV type and how the particles are formed. In both HPV type 6- and 11-positive cases, L1 IHC was strongly positive using light microscopy in the superficial layers of the tumour, and these cells were seen to be filled with microparticles in the nucleus using FE-SEM (Fig. 4A,B,G,H). In the slightly lower layers, in both HPV types 6- and 11-positive cases, L1 IHC was faintly positive by light microscopy, and particle structures in the nucleus were reduced in number and indistinct (Fig. 4A,C,G,I). No L1 IHC-positive cells were found in the granular, spinous, and basal layer using light microscopy, and no microparticle structures were observed in the nucleus (Fig. 4A,D–G,J–L). No morphological differences in the microparticles were observed between HPV types 6 and 11. In the HPV-negative case, no L1 IHC-positive cells were found using light microscopy, and no microparticle structures were observed in the nuclei (Fig. 4M–R).

### Association between L1 IHC and clinical severity

**Figure 5:**
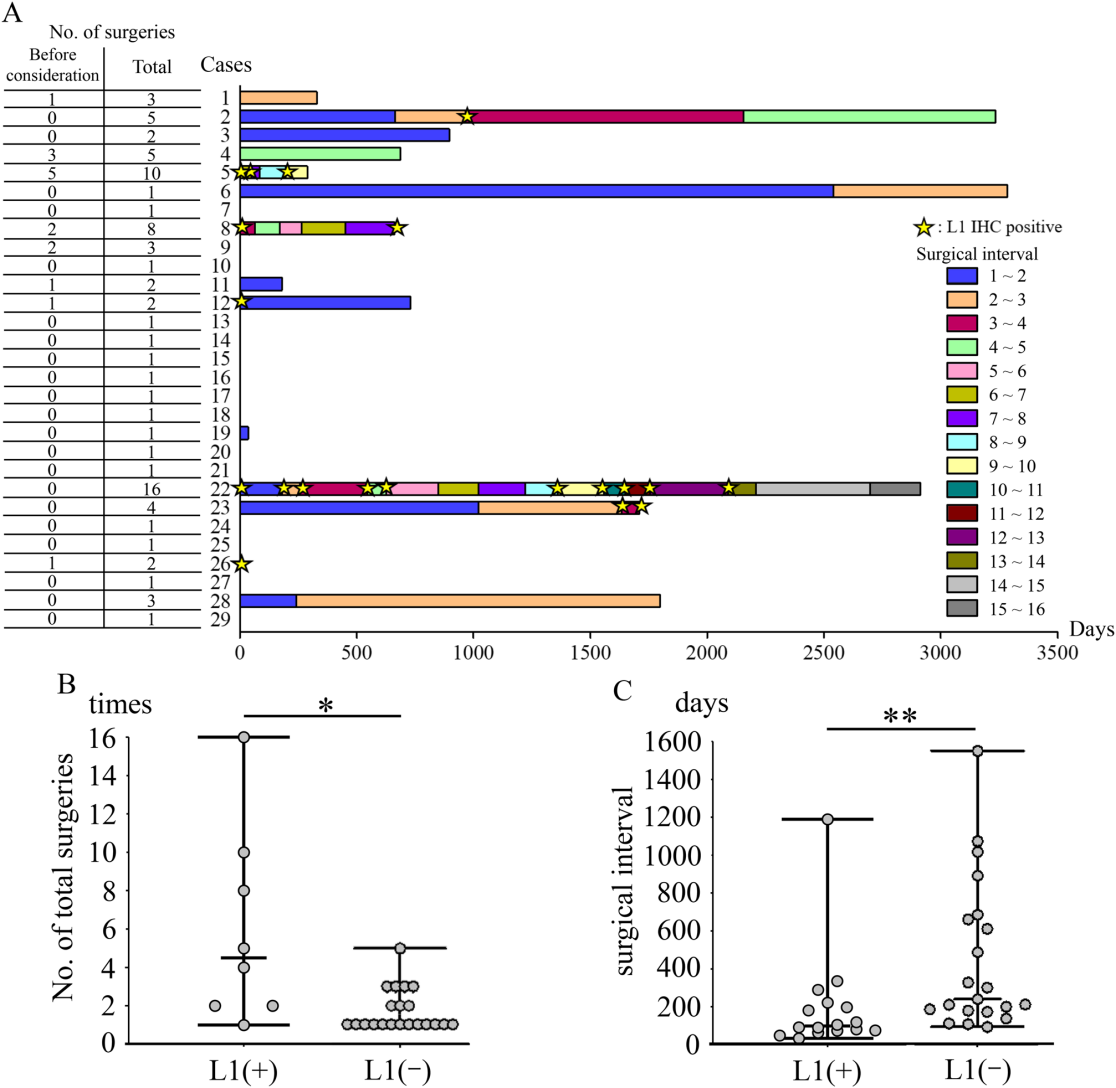
Association between L1 IHC and clinical severity. (**A**) HPV L1 IHC is positive in certain cases, with a high number of surgeries and short surgical intervals. The vertical axis indicates the number of cases, and the horizontal axis indicates the number of days. Colour of the bar indicates the surgical interval timing and the length represents each surgical interval. Stars indicate HPV L1 IHC positivity. (**B**) The total number of surgeries is significantly higher in L1 IHC-positive cases (*p* = 0.04). (**C**) The interval between surgeries is significantly shorter in L1 IHC-positive cases (*p* < 0.01). *Indicates *p* < 0.05 and ** indicates *p* < 0.01. *HPV* Human papillomavirus, *IHC* Immunohistochemistry.

We next examined the clinical severity of L1 IHC-positive cases, which are characterised by active HPV particle production. In Fig. 5A, the colour of the bar indicates the surgical interval timing and the length represents each surgical interval. The star indicates positive L1 IHC. The number of cases that were L1 IHC-positive was unevenly distributed, the number of surgeries was high, and the surgical interval was short (Fig. 5A). The number of surgeries was significantly higher in L1 IHC-positive patients (*p* = 0.04) (Fig. 5B). Furthermore, the surgical interval was significantly shorter in L1 IHC-positive patients (*p* < 0.01) (Fig. 5C).

### Assessment of HPV viral load and physical status

**Figure 6:**
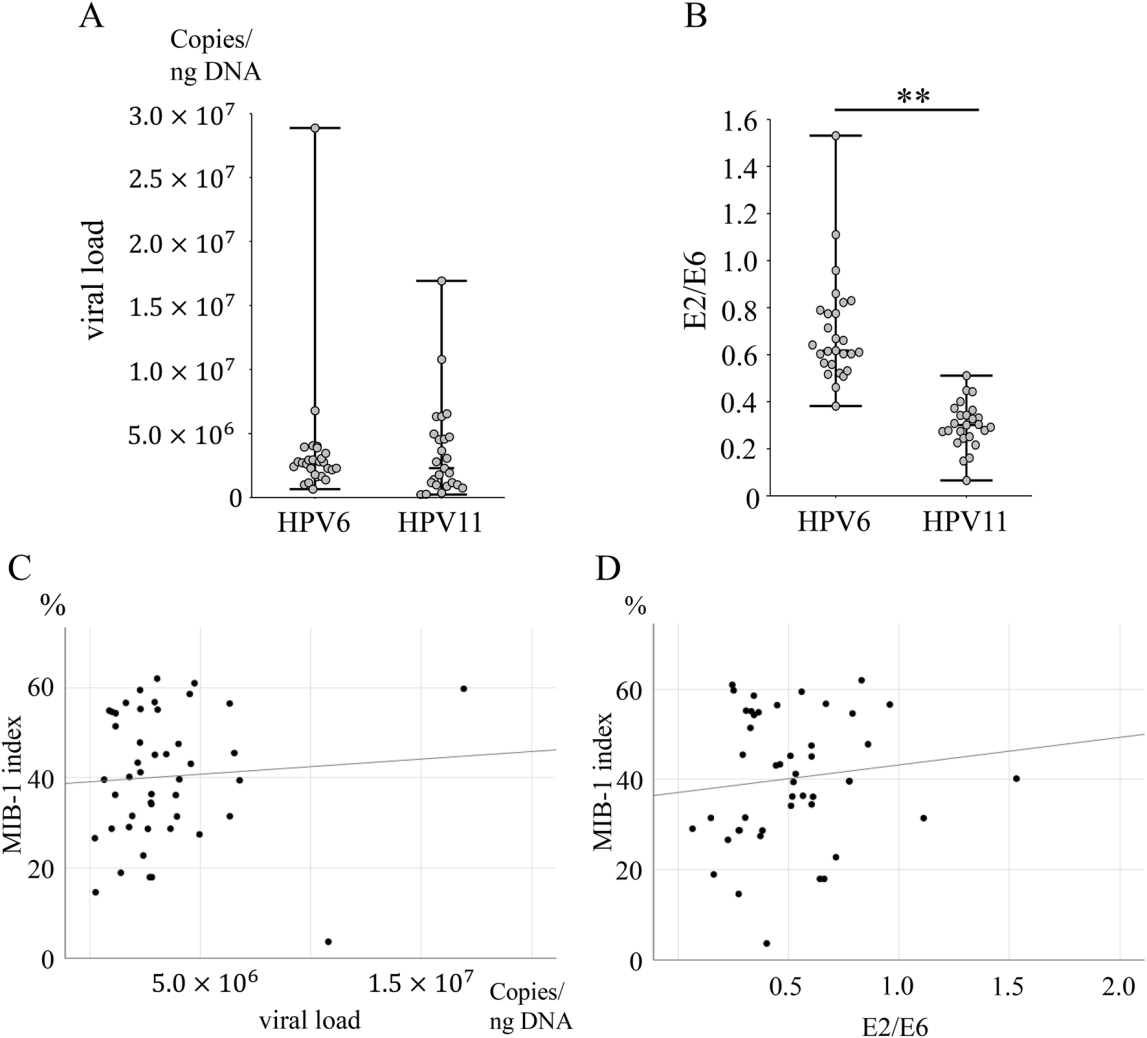
Assessment of HPV viral load and physical status. (**A**) Viral load shows no difference between HPV types 6 and 11 (*p* = 0.96). (**B**) HPV type 11 levels are significantly lower in E2/E6 (*p* < 0.01). (**C**) Viral load shows no correlation with the MIB-1 index (r = 0.07, *p* = 0.66). (**D**) E2/E6 shows no correlation with the MIB-1 index (r = 0.12, *p* = 0.44). **Indicates *p* < 0.01. *HPV* Human papillomavirus.

Next, we examined viral load and physical status. HPV viral load did not differ between HPV types 6 and 11 (*p* = 0.96) (Fig. 6A), and the number of HPV type 11 cases was significantly lower in E2/E6 (*p* < 0.01) (Fig. 6B). This result indicates that HPV type 11 is more integrated than type 6. HPV viral load and E2/E6 showed no correlation with the MIB-1 index (r = 0.07, *p* = 0.66, and r = 0.12, *p* = 0.44) (Fig. 6C,D).

### Derkay score and its relationship with IHC, HPV viral load, physical status, and surgical interval

**Figure 7:**
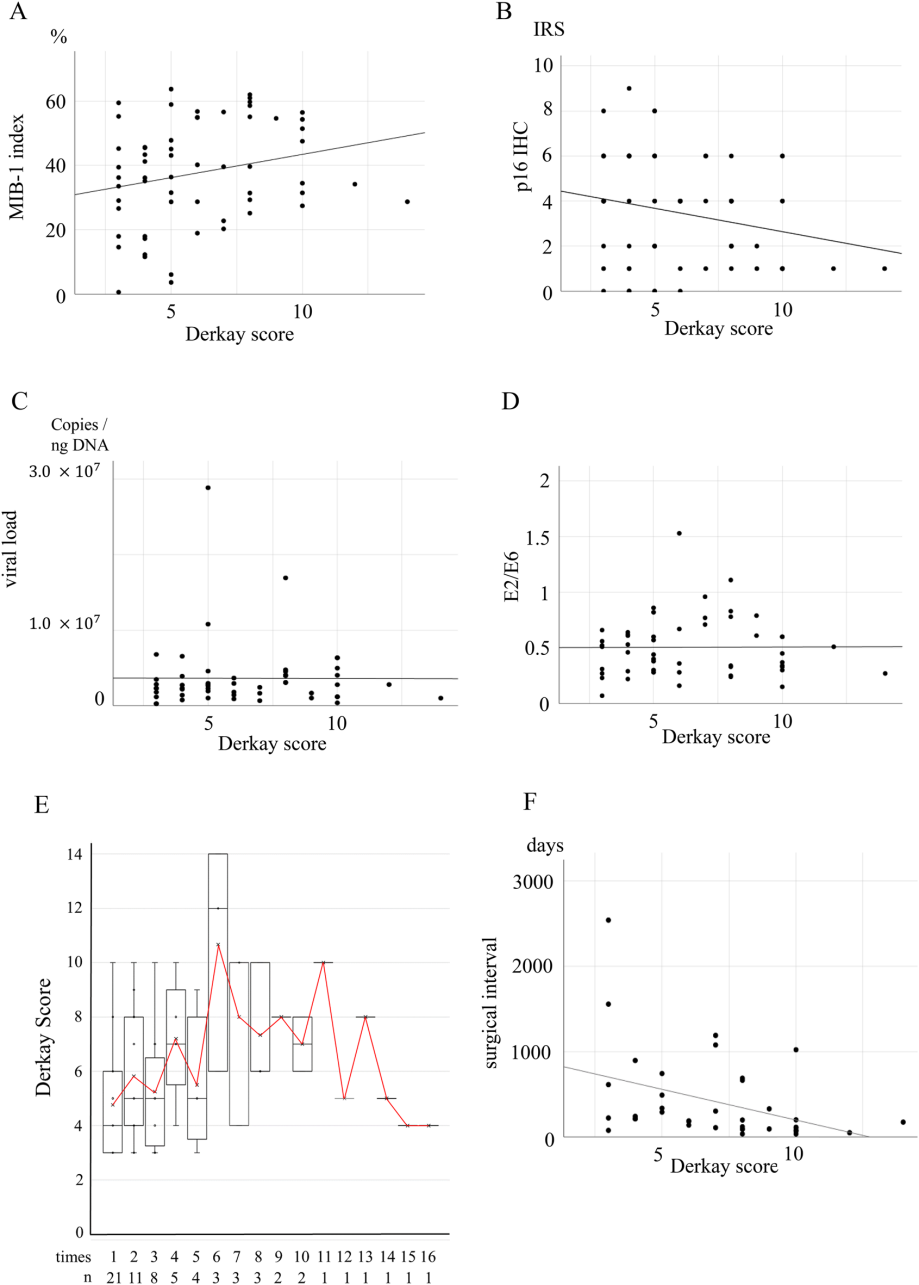
Scatter plots and regression lines for the Derkay score. (**A**) MIB-1 index and Derkay score show a weak positive correlation (r = 0.23, *p* = 0.07). (**B**) P16 IHC and Derkay scores show a weak negative correlation (r =  − 0.23, *p* = 0.06). (**C**) Viral load and Derkay scores show no correlation (r =  − 0.004, *p* = 0.976). (**D**) E2/E6 and Derkay scores show no correlation (r = 0.006, *p* = 0.966). (**E**) There is no significant trend in the mean change in the Derkay Score at each time of surgery. (**F**) Surgical interval and Derkay score show a negative correlation (r =  − 0.40, *p* = 0.01). *IHC* Immunohistochemistry, *DS* Derkay score.

We also evaluated the correlation between the Derkay score and IHC, HPV viral load, physical status, and surgical interval. L1 IHC-positivity was defined as 1 and negative as 0. L1 IHC positivity was associated with a high Derkay score (odds ratio: 1.28, 95% confidence interval: 1.04–1.57, *p* = 0.02). The MIB-1 index and Derkay score, a measure of severity, displayed a weak positive correlation (r = 0.23, *p* = 0.07) (Fig. 7A). In contrast, p16 IHC, a surrogate marker for HPV in oropharyngeal carcinoma, and Derkay scores exhibited a weak negative correlation (r =  − 0.23, *p* = 0.06) (Fig. 7B). Since L1 IHC-positivity and Derkay score were associated, we expected HPV viral load and E2/E6 to be correlated with Derkay score; however, no correlation was observed (r =  − 0.004, *p* = 0.976 and r = 0.006, *p* = 0.966, respectively) (Fig. 7C,D).

No significant trend was observed in the mean change in the Derkay score at each time of surgery (Fig. 7E). Surgical interval and Derkay score displayed a negative correlation (r =  − 0.40, *p* = 0.01) (Fig. 7F). This shows that the surgical interval could be a useful indicator of severity.

## Discussion

HPV is a double-stranded DNA virus approximately 50–60 nm in size without an envelope^23^. Since HPV DNA is detected in the laryngeal mucosa in remission, persistent infection is thought to be the pathogenesis of RRP^13^. In this study, IHC revealed that L1, which is a capsid protein, and E4, which is involved in HPV particle formation^24,25^, were correlated in the RRP tissue. Furthermore, observation of L1 IHC-positive cells by NanoSuit-CLEM and TEM revealed that the nuclei were filled with microparticles approximately 50–60 nm in size, while similar findings were not observed in L1 IHC-negative cell nuclei, indicating that these particles were HPV particles. Furthermore, this study suggests that HPV particles begin to form the tumour surface layer and increase in number as they move upwards. This study proved for the first time that HPV particles are continuously produced in severe RRP, which may result in persistent infection. In other words, we successfully visualised part of the HPV lifecycle in the RRP with FFPE sections.

This study also focused on HPV involvement. The HPV frequency is reportedly more than 80% based on recent papers on RRP^4–6^, but a few reports demonstrated an HPV involvement of approximately 60%^1,2^. Reports also exist where clinical diagnosis has been made without examining whether or not HPV is involved^7–9^. We examined HPV by PCR, L1 IHC, E4 IHC, and qPCR for E2/E6, all of which are undetectable in HPV-negative cases. These results indicate the presence of HPV-negative cases even in pathological cases of papilloma clinically diagnosed as RRP. In adult onset LP, the age group was lower for HPV-positive cases. Genetic mutations in *RAS* have been reported in HPV-negative cases^26^. They could be older since genetic mutation is the primary aetiology. The present data also showed a trend towards older age in HPV-negative RRP. On the other hand, the age of HPV types 6- and 11-positive RRP was not different in many reports; hence, the significant difference in this study could be attributed to the small sample size.

Owing to the wide range of severity of RRP, determining disease severity is crucial. The Derkay score is a widely used assessment tool. A study examining 721 patients with juvenile-onset RRP reported that a high Derkay score was a risk factor for recurrence^27^. However, two limitations of the Derkay score are that the score cannot predict the site of recurrence, and the score could vary from evaluator to evaluator since no clear criteria exist for tumour size or other factors. Another well-known risk factor is the involvement of HPV type 11^27–29^. In this study, the number of surgeries did not differ between HPV types 6 and 11; however, there was a statistically significant difference between HPV-positive and HPV-negative patients. Furthermore, we found that the surgical interval was shorter, and the number of surgeries was higher in L1 IHC-positive cases, which indicates active HPV particle production. These findings suggest that persistent production of HPV particles, resulting in persistent infection, is the main pathogenesis of RRP. The combination of surgery and HPV vaccination has been reported to reduce the risk of recurrence^30,31^. These reports support this hypothesis. Time to diagnosis could be a confounding factor. However, looking at the L1 IHC-positive cases in this study, they tend to be positive regardless of the surgeries performed (Fig. 5A and Supplementary Table S1, Case 5, 8, 22, 23). In addition, even short surgical intervals can occur with L1 IHC-positivity. Thus, patient factors, such as the immune response, could be more relevant than time. Furthermore, we found that L1 IHC positivity was associated with a high Derkay score; however, L1 IHC and Derkay score could evaluate different aspects. Derkay score is unlikely to be considered high without multiple lesions and cannot evaluate the pathophysiology of persistent HPV infection. Moreover, L1 IHC is not able to evaluate the entire tumour because it only evaluates the tissue on the glass slide; however, it demonstrates strong evidence of active HPV particle production. Thus, the Derkay score and HPV particle formation could evaluate different aspects of RRP. In contrast, a weak negative correlation was observed between the Derkay score and p16 IHC, a known surrogate marker for HPV^32,33^. Therefore, we suggest testing for L1 IHC in addition to the Derkay score to accurately assess RRP severity.

We also examined the viral load and physical status. Viral load did not differ between HPV types 6 and 11, but HPV type 11 was integrated-type dominant. In contrast, viral load and physical status did not correlate with the MIB-1 index. In addition, viral load and physical status did not correlate with the Derkay score. Previous reports revealed that viral load decreased progressively with each surgery until remission was achieved^34^, while others showed that viral load and physical status are not related to tumour volume^22^; hence, no consensus exists. Viral load varied widely within the same case. Viral load may vary based on the site being sampled. However, since samples were not taken separately for each subsite, we were unable to examine this point. Further research is required to elucidate these findings; however, viral load and physical status do not appear to be indicators of clinical severity.

We identified HPV particles in FFPE sections, and the observation of HPV particles was associated with RRP severity. However, this study had several limitations. The number of cases in this study was small, although 29 cases may be considered a relatively large sample for an RRP study. This study did not include juvenile onset RRP, HPV-vaccinated patients, and lower respiratory tract lesions; thus, whether L1 IHC is effective in such cases remains unclear. Although recurrence interval rather than surgical interval seems to be a better indicator, surgical interval was used as an indicator in this study since accurately determining the recurrence timing was challenging. However, since the surgical interval correlates with the Derkay score, it can be a useful indicator of severity. All the patients with malignant transformation were HPV-negative. A report from Taiwan suggested that HPV-negative status is a risk factor for malignant transformation^35^. Therefore, although HPV-negative status could be a risk factor for malignant transformation, we did not study this point in detail.

In conclusion, using NanoSuit-CLEM and TEM, we succeeded for the first time in visualising a part of the HPV lifecycle of RRP in FFPE sections. Persistent infection with HPV particles due to continuous HPV particle production is correlated with clinical severity. L1 IHC is important for evaluating the severity of RRP in addition to the Derkay score.

## Methods

### Tumour samples

Sixty-eight RRP samples were obtained from 29 patients who underwent surgery at the Hamamatsu University School of Medicine, Seirei Hamamatsu General Hospital, Seirei Mikatahara General Hospital, and Fujieda Municipal General Hospital. Samples were obtained from September 2005 to June 2021. All cases were pathologically diagnosed as papilloma before they were included in the study. In all cases, total tumour removal was performed each time, and the cases where tumour was left were excluded. Whenever possible, surgery was performed within a few months after observing recurrence. The study protocol was approved by the Institutional Review Board of Hamamatsu University School of Medicine (approval number: 19–222). All methods were performed in accordance with the Declaration of Helsinki. Written informed consent was obtained from all patients. Medical information, including patient age, sex, alcohol exposure, smoking status, Derkay score, and date of surgery, was retrieved from patient records.

The Derkay Score is a widely used severity assessment method. The formula of the Derkay score is ‘the sum of the points calculated from the size of the tumour at each site’ plus ‘the points calculated from the clinical findings’. The tumour size assessment is based on the aerodigestive tract divided into 25 subsites, each of which is given a score of 0 to 3 (0 = no lesion, 1 = surface lesion, 2 = raised lesion, and 3 = bulky lesion). The clinical findings assessment are based on the patient’s voice (0 = normal, 1 = abnormal, and 2 = aphonic), patient’s stridor (0 = absent, 1 = present with activity, and 2 = present at rest), urgency of today’s intervention (0 = scheduled, 1 = elective, 2 = urgent, and 3 = emergent), and level of respiratory distress (0 = none, 1 = mild, 2 = moderate, 3 = severe, and 4 = extreme)^12^.

### DNA extraction, HPV detection, and qPCR analysis of viral load and physical status

Tissue for DNA extraction was sampled from the area with the largest tumour volume. In cases where tissue sampling for this study was inadequate, DNA was extracted from FFPE tissue. DNA was extracted using a QIAamp DNA Mini Kit or QIAamp DNA FFPE Tissue Kit (Qiagen, Hilden, Germany). The TaKaRa PCR Human Papillomavirus Typing Set (Takara Bio, Kusatsu, Japan) primer set was used to detect HPV. Samples that were detected as HPV-positive by PCR were subjected to direct sequencing analysis using a 3500xL Genetic Analyzer (Applied Biosystems, Waltham, MA, USA). HPV type was determined by searching for sequences using BLAST (https://blast.ncbi.nlm.nih.gov/).

The viral load of HPV type 6 or 11 and the physical status were evaluated by qPCR using the StepOne Real-Time PCR System (Applied Biosystems, Waltham, MA, USA). Primers for HPV type 6 and 11 specific E2 and E6, and actin beta were designed (Supplementary Table S2). Standard curves for E2, E6, and actin beta were set using a cloning plasmid as described previously^21,22,34^. The E6 copy number per nanogram of DNA was defined as the viral load. The ratio of E2 copy number/E6 copy number represents the physical status of HPV. E2/E6 < 1 indicates that both integrated and episomal (mixed) forms exist, E2/E6 ≥ 1 indicates the predominance of episomal form, and E2/E6 = 0 indicates an integrated form only^36^.

### Immunohistochemistry

FFPE tissue sections (4-μm thick) were prepared. Antigen retrieval was performed using the Epitope Retrieval Solution pH 9 (Leica Biosystems, Wetzlar, Germany). To block endogenous peroxidase activity, the sections were incubated in a 0.3% H_2_O_2_ solution in methanol. Ten percent goat serum (Nichirei Bioscience, Tokyo, Japan) was used to block the sections. The primary antibodies used were HPV-L1 antibody (K1H8) (Thermo Fisher Scientific, Waltham, MA, USA), p16 (E4H6) (Roche, Basel, Switzerland), and Ki-67 (MIB-1) (DAKO, Santa Clara, CA, USA). HPV type 6 E4 and type 11 E4 antibodies, as described previously, were also used^21^. For secondary antibodies, a MAX-PO(M) or MAX-PO(R) kit (Nichirei Bioscience, Tokyo, Japan) was used. The sections were visualised with DAB.

L1 and E4 double staining was performed by L1 and visualised by DAB, and E4 was visualised using the ImmPACT Vector Red AP Substrate Kit (Vector Laboratories, Newark, CA, USA).

IHC evaluations were performed independently by two pathologists who were blinded to patient information. IHC was evaluated as positive or negative for L1 and E4, the MIB-1 index was calculated as percentage of Ki-67 IHC-positive cells/total tumour cells, and p16 was calculated as the immunoreactive score (IRS) using staining intensity as percentage of the positive tumour cells (Suppl. Fig. S2)^37^.

### *RNA *in situ* hybridisation*

HPV type 6 E4 and type 11 E4 probes were used. The protocol was described previously^21,22^. Briefly, digoxigenin-labelled probes were prepared and hybridised overnight at 60 °C. Detection was performed by nitroblue tetrazolium/5-bromo- 4-chloro-3-indolyl phosphate solution.

### HPV particle detection using the NanoSuit-CLEM method (FE-SEM observation)

IHC with L1 antibody was performed (DAB-stained). Next, images of the areas to be observed by FE-SEM were obtained. The slides were incubated with 1% osmium solution for 5 min. Osmium reacts specifically with DAB and is enhanced when observed using FE-SEM^38^. The osmium vapor deposition was performed using an osmium coater (HPC-1SW) (Vacuum device, Mito, Japan). Hitachi S-4800 Field Emission Scanning Electron Microscope (Hitachi, Tokyo, Japan) was used for FE-SEM observations and operated at an acceleration voltage of 5.0 kV. The images were captured in the yttrium–aluminum-garnet backscattered electron (YAG-BSE) mode.

### Field of view evaluation of the FE-SEM observation area using TEM

FFPE tissue Sects. (8-μm thick) were prepared using non-coated glass. The NanoSuit Solution II (Nisshin EM, Tokyo, Japan) was applied to the slides, which were rotated 3000 rpm by spin coater to create a nanofilm. Then, FE-SEM observations were performed. The FFPE tissue sections on slide glass were re-fixed with 2% glutaraldehyde and 2% osmium tetroxide, and embedded in epoxy resin (Nisshin EM, Tokyo, Japan). Blocks (0.5 mm^2^) were cut out to unify the FE-SEM observation area. Ultrathin slides (60–80 nm) were prepared and stained with lead acetate and uranium acetate. JEM-1400 (JEOL, Tokyo, Japan) was used for the transmission electron microscopy (TEM) observations.

### Statistical analysis

Data for the two populations in the two categories were subjected to Fisher’s exact test. Fisher’s exact test was conducted using two-tailed tests. The relevant data included sex, smoking, alcohol, and malignant transformation. Welch’s t-test was applied to compare the two sets of data. A Welch’s t-test was conducted using two-tailed tests. The relevant data included age, total number of surgeries, Derkay score, IHC data, and viral load. Since these data did not show homogeneity of variance, Welch’s t-test was performed. The Pearson product-moment correlation coefficient was used to assess the strength of the linear relationship between the two data or random variables. A binomial logistic regression analysis was applied for determining the association between L1 IHC and the Derkay Score. Statistical significance was set at *p* < 0.05. Statistical analyses were performed using SPSS ver.26 (IBM Corp., Armonk, NY, USA) and KyPlot ver.6.0.2 (KyensLab, Tokyo, Japan).

## Supplementary Information

Supplementary Information.

## Data Availability

All data generated or analysed during this study are included in this published article and its Supplementary Information files.
