# Supplementary material for: Association between human papillomavirus particle production and the severity of recurrent respiratory papillomatosis
Source: Sci Rep. 2023 Apr 6;13:5514. doi: 10.1038/s41598-023-32486-8 (PMC10079853; doi:10.1038/s41598-023-32486-8)
Supplement: Supplementary file 1 — Supplementary Information. [file 41598_2023_32486_MOESM1_ESM.pdf]

# Association between Human Papillomavirus Particle Production and the Severity of Recurrent Respiratory Papillomatosis

Satoshi Yamada[1, 2]; Toshiya Itoh[2,3]; Taro Ikegami[4]; Atsushi Imai[1]; Daiki Mochizuki[1]; Hiroshi Nakanishi[1]; Ryuji Ishikawa[1]; Junya Kita[1]; Yuki Nakamura[1]; Yoshinori Takizawa[1]; Jun Okamura[5]; Yoshihiro Noda[6]; Toshihide Iwashita[7]; Takahiko Hariyama[2]; Mikio Suzuki[4]; Kiyoshi Misawa*[1]; Hideya Kawasaki*[2,7]

[1] Department of Otolaryngology/Head and Neck Surgery, Hamamatsu University School of Medicine, Hamamatsu, Japan

[2] Preeminent Medical Photonics Education and Research Center Institute for NanoSuit Research, Hamamatsu University School of Medicine, Hamamatsu, Japan

[3] Department of Obstetrics & Gynecology, Hamamatsu University School of Medicine, Hamamatsu, Japan

[4] Department of Otorhinolaryngology, Head and Neck Surgery, Graduate School of Medicine, University of the Ryukyus, Okinawa, Japan

[5] Department of Otorhinolaryngology, Seirei Hamamatsu General Hospital, Hamamatsu, Japan.

[6] Department of Otorhinolaryngology, Seirei Mikatahara General Hospital, Hamamatsu, Japan

[7] Department of Regenerative & Infectious Pathology, Hamamatsu University School of Medicine, Hamamatsu, Japan

**Supplemental Figure S1. HPV sequencing analysis**

Case No. 9 HPV type 6

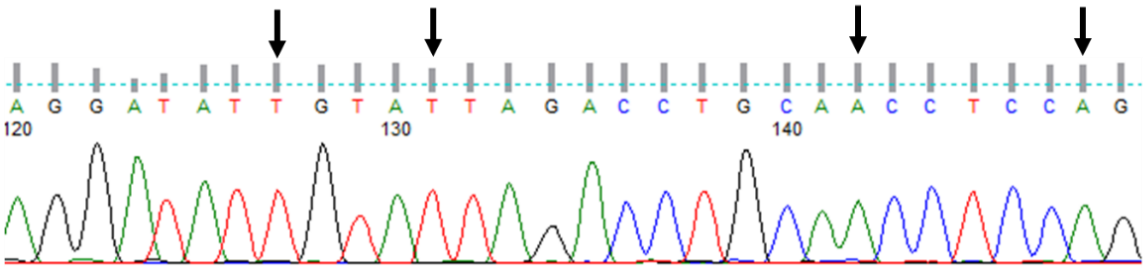


Case No. 11 HPV type 11

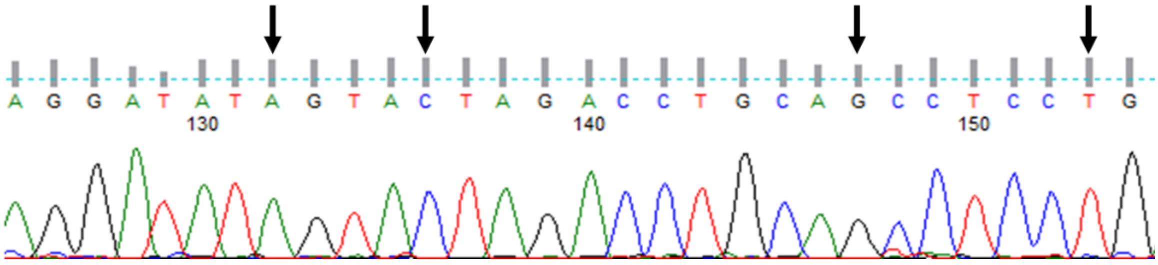


HPV type 6 and HPV type 11 sequencing results. Arrows indicate different nucleotide sequences.
HPV: human papillomavirus

**Supplemental Figure S2. Immunoreactive score**

| Positive tumor cells \ Intensity | Weak Staining = 1 point | Moderate Staining = 2 points | Strong Staining = 3 points |
|---|---|---|---|
| < 10% = 1 point | IRS = 1 | IRS = 2 | IRS = 3 |
| 10 - 50% = 2 points | IRS = 2 | IRS = 4 | IRS = 6 |
| 51 - 80% = 3 points | IRS = 3 | IRS = 6 | IRS = 9 |
| > 80% = 4 points | IRS = 4 | IRS = 8 | IRS = 12 |

The immunoreactive score (IRS) is used to evaluate p16 IHC staining

IHC: immunohistochemistry

## Supplemental Table S1 Detailed data on patient characteristics

| Case No. | Operation No. from this study | Age | Sex | Smoking | Alcohol | HPV | Total operation No. | DS | Operation interval (days) | p16 IRS | L1 IHC | MIB-1 index | Viral load (Copies/ng DNA) | E2/E6 | MT |
|---|---|---|---|---|---|---|---|---|---|---|---|---|---|---|---|
| 1 | 1-1 | 43 | M | − | + | 6 | 3 | 9 | N.A. | 2 | − | 54.62% | $1.00 \times 10^6$ | 0.79 | − |
| | 1-2 | | | | | | | 3 | 329 | 0 | − | 36.22% | $1.15 \times 10^6$ | 0.52 | − |
| 2 | 2-1 | 30 | M | + | + | 6 | 5 | 8 | − | 6 | − | 31.42% | $3.94 \times 10^6$ | 1.11 | − |
| | 2-2 | | | | | | | 7 | 664 | 6 | − | 22.77% | $2.42 \times 10^6$ | 0.71 | − |
| | 2-3 | | | | | | | 7 | 302 | 6 | + | 39.59% | $6.48 \times 10^5$ | 0.77 | − |
| | 2-4 | | | | | | | 7 | 1190 | 4 | − | 20.28% | N.A. | N.A. | − |
| | 2-5 | | | | | | | 5 | 1078 | 2 | − | 36.38% | $2.79 \times 10^6$ | 0.57 | − |
| 3 | 3-1 | 42 | M | + | + | 6 | 2 | 4 | − | 0 | − | 17.95% | $2.72 \times 10^6$ | 0.64 | − |
| | 3-2 | | | | | | | 5 | 896 | 2 | − | 28.68% | $2.63 \times 10^6$ | 0.38 | − |
| 4 | 4-1 | 57 | M | + | + | 6 | 5 | 8 | N.A. | 2 | − | 39.66% | $4.05 \times 10^6$ | 0.78 | − |
| | 4-2 | | | | | | | 5 | 688 | 6 | − | 47.81% | $2.27 \times 10^6$ | 0.86 | − |
| 5 | 5-1 | 47 | M | + | + | 11 | 10 | 12 | N.A. | 1 | + | 34.16% | $2.79 \times 10^6$ | 0.51 | − |
| | 5-2 | | | | | | | 10 | 49 | 1 | + | 27.44% | $4.96 \times 10^6$ | 0.37 | − |
| | 5-3 | | | | | | | 10 | 34 | 1 | − | 31.47% | $6.34 \times 10^6$ | 0.15 | − |
| | 5-4 | | | | | | | 8 | 113 | 2 | + | 58.57% | $4.52 \times 10^6$ | 0.34 | − |
| | 5-5 | | | | | | | 6 | 92 | 4 | − | 18.94% | $1.40 \times 10^6$ | 0.16 | − |
| 6 | 6-1 | 70 | M | + | + | − | 3 | 3 | − | 1 | − | 0.60% | − | − | − |
| | 6-2 | | | | | | | 5 | 2542 | 0 | − | 58.93% | − | − | − |
| | 6-3 | | | | | | | 5 | 743 | 0 | − | 63.71% | − | − | + |
| 7 | 7-1 | 54 | F | − | − | − | 1 | 6 | − | 0 | − | 54.93% | − | − | + |
| 8 | 8-1 | 52 | M | − | + | 6 | 8 | 10 | N.A. | 4 | + | 47.51% | $4.01 \times 10^6$ | 0.60 | − |
| | 8-2 | | | | | | | 7 | 63 | 1 | − | 56.60% | $1.63 \times 10^6$ | 0.96 | − |
| | 8-3 | | | | | | | 9 | 108 | 1 | − | N.A. | $1.66 \times 10^6$ | 0.61 | − |
| | 8-4 | | | | | | | 6 | 93 | 1 | − | 40.19% | $1.79 \times 10^6$ | 1.53 | − |
| | 8-5 | | | | | | | 4 | 187 | 0 | − | N.A. | $1.38 \times 10^6$ | 0.62 | − |
| | 8-6 | | | | | | | 6 | 211 | 4 | + | 56.77% | $2.93 \times 10^6$ | 0.67 | − |
| 9 | 9-1 | 46 | M | + | + | 6 | 3 | 5 | N.A. | 6 | − | 45.10% | $2.95 \times 10^6$ | 0.60 | − |
| 10 | 10-1 | 45 | F | + | − | 6 | 1 | 3 | − | 4 | − | 17.95% | $2.81 \times 10^6$ | 0.66 | − |

| | | | | | | | | | | | | | | | |
|---|---|---|---|---|---|---|---|---|---|---|---|---|---|---|---|
| 11 | 11-1 | 42 | M | + | − | − | 2 | 5 | 180 | 2 | − | 6.08% | − | − | − |
| 12 | 12-1 | 43 | M | + | + | 6 | 2 | 5 | 732 | 4 | + | N.A. | $2.89 \times 10^7$ | 0.82 | − |
| 13 | 13-1 | 49 | M | + | − | 6 | 1 | 3 | − | 4 | − | N.A. | − | − | − |
| 14 | 14-1 | 69 | M | + | + | 11 | 1 | 3 | − | 8 | − | 26.61% | $2.29 \times 10^5$ | 0.23 | − |
| 15 | 15-1 | 66 | F | − | − | 11 | 1 | 3 | − | 6 | − | 29.07% | $1.78 \times 10^6$ | 0.07 | − |
| 16 | 16-1 | 75 | M | + | + | 11 | 1 | 3 | − | 2 | − | 14.61% | $2.61 \times 10^5$ | 0.27 | − |
| 17 | 17-1 | 70 | M | + | + | − | 1 | 3 | − | 6 | − | 33.53% | − | − | − |
| 18 | 18-1 | 50 | M | + | + | 11 | 1 | 5 | − | 8 | − | 3.65% | $1.08 \times 10^7$ | 0.40 | − |
| 19 | 19-1 | 86 | M | + | + | − | 2 | 8 | − | 1 | − | 29.31% | − | − | − |
| | 19-2 | | | | | | | 4 | 35 | 1 | − | 12.28% | − | − | − |
| 20 | 20-1 | 47 | M | + | + | 11 | 1 | 4 | − | 6 | + | 35.17% | N.A. | N.A. | − |
| 21 | 21-1 | 44 | M | − | + | − | 1 | 4 | − | 4 | − | 17.25% | − | − | − |
| 22 | 22-1 | 45 | M | + | + | 11 | 16 | 6 | − | 4 | + | 28.71% | $3.65 \times 10^6$ | 0.28 | − |
| | 22-2 | | | | | | | 10 | 182 | 4 | + | 56.46% | $6.33 \times 10^6$ | 0.45 | − |
| | 22-3 | | | | | | | 5 | 74 | 8 | + | 43.10% | $4.57 \times 10^6$ | 0.44 | − |
| | 22-4 | | | | | | | 10 | 290 | 6 | + | 54.30% | $1.17 \times 10^6$ | 0.34 | − |
| | 22-5 | | | | | | | 3 | 81 | 4 | + | 55.24% | $2.29 \times 10^6$ | 0.31 | − |
| | 22-6 | | | | | | | 14 | 222 | 1 | − | 28.72% | $9.87 \times 10^6$ | 0.27 | − |
| | 22-7 | | | | | | | 10 | 173 | 1 | − | N.A. | $3.58 \times 10^5$ | 0.30 | − |
| | 22-8 | | | | | | | 6 | 200 | 4 | − | 54.88% | $8.80 \times 10^5$ | 0.36 | − |
| | 22-9 | | | | | | | 8 | 138 | 4 | + | 60.96% | $4.74 \times 10^6$ | 0.24 | − |
| | 22-10 | | | | | | | 8 | 198 | 4 | + | 55.11% | $3.07 \times 10^6$ | 0.33 | − |
| | 22-11 | | | | | | | 10 | 91 | 1 | + | 51.42% | $1.17 \times 10^6$ | 0.33 | − |
| | 22-12 | | | | | | | 5 | 105 | 4 | + | 31.55% | $1.92 \times 10^6$ | 0.30 | − |
| | 22-13 | | | | | | | 8 | 336 | 4 | + | 59.73% | $1.69 \times 10^7$ | 0.25 | − |
| | 22-14 | | | | | | | 5 | 119 | 6 | − | N.A. | $9.97 \times 10^5$ | 0.28 | − |
| | 22-15 | | | | | | | 4 | 490 | 9 | − | N.A. | $7.47 \times 10^5$ | 0.22 | − |
| | 22-16 | | | | | | | 4 | 213 | 1 | − | 45.48% | $6.54 \times 10^6$ | 0.29 | − |
| 23 | 23-1 | 58 | M | + | + | 6 | 4 | 10 | − | 6 | − | 34.47% | $2.76 \times 10^6$ | 0.60 | − |
| | 23-2 | | | | | | | 3 | 1022 | 4 | − | 59.45% | $2.27 \times 10^6$ | 0.56 | − |
| | 23-3 | | | | | | | 3 | 613 | 4 | + | 39.43% | $6.78 \times 10^6$ | 0.52 | − |
| | 23-4 | | | | | | | 4 | 76 | 6 | + | 36.16% | $3.89 \times 10^6$ | 0.61 | − |

| | | | | | | | | | | | | | | | |
|---|---|---|---|---|---|---|---|---|---|---|---|---|---|---|---|
| 24 | 24-1 | 59 | M | + | − | − | 1 | 8 | − | 2 | − | 25.16% | − | − | − |
| 25 | 25-1 | 37 | M | + | + | − | 1 | 4 | − | 2 | − | N.A. | − | − | − |
| 26 | 26-1 | 42 | M | + | + | 6 | 2 | 8 | N.A. | 4 | + | 61.98% | $3.05 \times 10^6$ | 0.83 | − |
| 27 | 27-1 | 62 | M | + | + | − | 1 | 4 | − | 6 | − | 45.63% | − | − | + |
| 28 | 28-1 | 31 | M | + | − | 6 | 3 | 4 | − | 6 | − | 43.35% | $2.17 \times 10^6$ | 0.46 | − |
| | 28-2 | | | | | | | 3 | 241 | 6 | − | 45.24% | $3.46 \times 10^6$ | 0.51 | − |
| | 28-3 | | | | | | | 4 | 1557 | 6 | − | 41.23% | $2.29 \times 10^6$ | 0.53 | − |
| 29 | 29-1 | 77 | M | + | + | − | 1 | 4 | − | 0 | − | 11.58% | − | − | − |

M: male, F: female, DS: Derkay score, IRS: immunoreactive score, MT: malignant transformation, N.A.: not available (due to missing data or insufficient samples)

**Supplemental Table S2. Real time PCR primers set**

| Real-time PCR primers | Sequence (5'-3') | bp |
|---|---|---|
| HPV6 E2-F | TGAGTATAGTATGGAACCGTGGA | 100 |
| HPV6 E2-R | CTTCTACAGTTTTGCCCCGT | |
| HPV6 E6-F | GCTACCTGTGTCACAAACCG | 100 |
| HPV6 E6-R | CAGCGACCCTTCCACGTA | |
| HPV11 E2-F | GACCGTCCACTAACAACACC | 102 |
| HPV11 E2-R | TCCTTCTTTGGTGCTTGTTGT | |
| HPV11 E6-F | CAAGCCGTTGTGTGAAATAGAA | 100 |
| HPV11 E6-R | CCAGCAGTGTAAGCAACGAC | |
| ACTB-F | TGCCCTCATTTCCCTCTCAG | 101 |
| ACTB-R | CGTACAGGTCTTTGCGGATG | |

PCR: polymerase chain reaction
